# Sex differences in the treatment and outcome of emergency general surgery

**DOI:** 10.1371/journal.pone.0224278

**Published:** 2019-11-04

**Authors:** Diana Rucker, Lindsey M. Warkentin, Hanhmi Huynh, Rachel G. Khadaroo

**Affiliations:** 1 Department of Medicine, Division of Geriatrics, University of Alberta, Edmonton, AB, Canada; 2 Department of Surgery, University of Alberta, Edmonton, AB, Canada; 3 Department of Critical Care Medicine, University of Alberta, Edmonton, AB, Canada; University of Pittsburgh, UNITED STATES

## Abstract

**Background:**

Sociodemographic characteristics, such as sex, have been shown to influence health care delivery. Acute care surgery models are effective in decreasing mortality and morbidity after emergency surgeries, but sex-based differences in delivery and outcomes have not been explored. Our objective was to explore sex associated differences in the patient characteristics and clinical outcomes of those admitted to emergency general surgery.

**Methods:**

A post-hoc analysis of 512 emergency general surgical patients admitted consecutively to two tertiary care hospitals in Alberta Canada, between April 1, 2014 and July 31, 2015. We measured associations between sex and patient demographics, pre-, intra- and post-operative delivery of care, as well as post-operative outcomes.

**Findings:**

Of those excluded from the analysis, older females were more likely to undergo conservative management compared to older men (41% vs 34%, p = 0.03). Overall, there were no differences between sexes for time from admission to surgery, time spent in surgery, overall complication rate, mortality, hospital length of stay, or discharge disposition. Women were more likely to have a cancer diagnosis [OR 4.12 (95% CI: 1.61–10.5), p = 0.003, adjusted for age], while men were more likely to receive hernia surgery [OR 2.33 (95% CI 1.35–4.02), p = 0.002, adjusted for age and Charlson Comorbidity Index]. Finally, men were more likely to have a major respiratory complication [OR 2.73 (95% CI: 1.19–6.24), p = 0.02, adjusted for age].

**Conclusions:**

Only two differences in peri and post-operative complications between sexes were noted, which suggests sex-based disparity in quality of care is limited once a decision has been made to operate. Future studies with larger databases are needed to corroborate our findings and investigate potential sex biases in surgical versus conservative management.

## Introduction

Differences in health care delivery and outcome between men and women have been documented in many areas of research including sepsis [1, 2], myocardial infarction [3–5], vascular surgery [6, 7] and hip arthroplasty [8]. Sex related differences have been associated with physiology [4] as well as societal biases [9–12]. Identifying these differences is crucial in not only optimizing health care outcomes but also minimizing existing disparity of access to care [13, 14].

In the general surgery setting, studies have shown conflicting findings of sex disparities. For instance, females are less often diagnosed then men with colon cancer through screening [15–17]; however, females also had faster rates of surgery despite having a higher rate of multivisceral resection [16]. In other areas of elective general surgery, women were less likely to undergo laparoscopic repair of groin hernias than men in elective surgery setting [18].

Acute care surgery is a relatively newer surgical specialty that encompasses trauma, critical care and emergency general surgery (EGS) such as appendectomies, cholecystectomies, gastrointestinal obstruction release or resection, perforation repair, and emergency cancer surgery. The goal of EGS is to provide surgical care within the first 24 hours of hospitalization. Few studies have examined differences in sex in the EGS setting. Two studies that both used data from the Nationwide Inpatient Sample have noted that females undergo EGS more frequently [19]; however, when patients over 65 years of age were excluded, men were more likely to undergo EGS [20]. Neither paper assessed sex related differences in perioperative care or outcomes.

Exploring sex disparity is crucial to examining the models of surgical disease in men and women and expanding our understanding of health determinants for each sub-population. We sought to identify if there were sex differences within an EGS system for pre-, intra- and post-operative delivery of care, as well as post-operative outcomes.

## Methods

**Fig 1:**
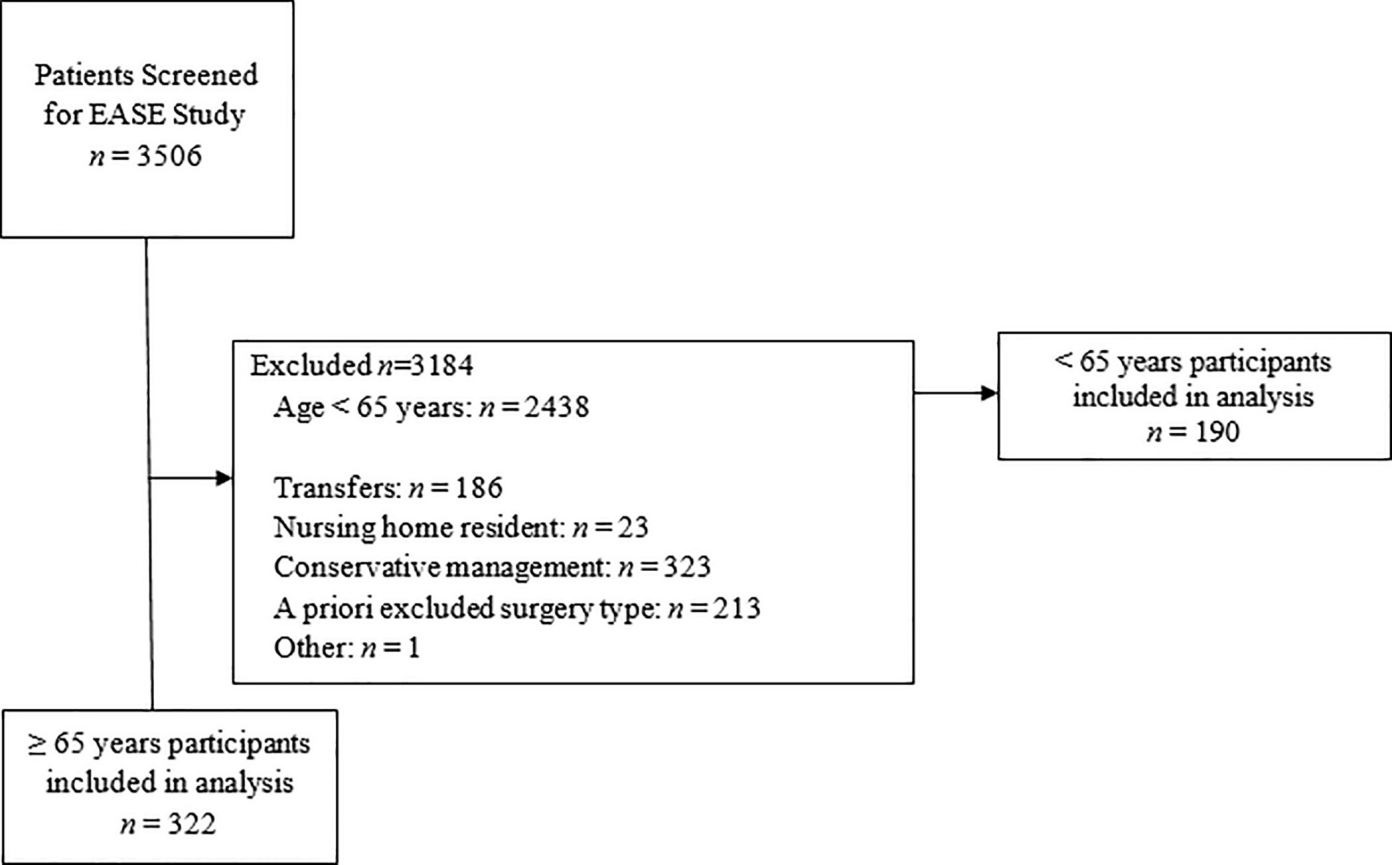
Selection of post-hoc analysis cohort.

All screened patients for the Elder-friendly Approaches to the Surgical Environment (EASE) study [21] were reviewed for inclusion in this post-hoc analysis. Elderly participants (≥ 65 years old) that were prospectively enrolled through the EASE study prior to the implementation of the care intervention were included; a convenience comparison cohort of younger patients (< 65 years old)–who otherwise would have been eligible for the elder-friendly study except for age–were also included (Fig 1). Participants were admitted from two tertiary care centers in Edmonton, Alberta and Calgary, Alberta, Canada. Patients were included in this post-hoc analysis if they underwent emergency abdominal surgery between April 1, 2014 and July 31, 2015. Patients were hierarchically excluded if they were considered to be the following cases: 1) transferred from another hospital or ward 2) nursing home resident requiring full nursing care on admission, 3) received conservative management 4) surgical cases that were elective, palliative, trauma and/or non-abdominal.

All screened patients had age, sex and reason for inclusion and/or exclusion from the EASE study recorded. Trained research assistants collected pre-, intra- and post-operative details for those eligible for post-hoc analysis. Details included: patient demographics (age, body mass index, Charlson Comorbidity Index, ASA Physical System classification, number of medications, admitting diagnosis); pre-, intra- and post-operative delivery of care (admission location, time waiting until admission, time waiting until surgery, number of surgeries, surgery type, time in the operating room, disposition after surgery, time until first mobilization, foley catheter and total parenteral nutrition use); and post-operative outcomes (complications defined using ICD-10 codes [22] and classified using Clavian-Dindo [23] as minor (Grade I and II) and major (Grade III and IV), in-hospital death, length of hospital stay, discharge status).

### Statistical analyses

Given the exploratory nature of this analysis, missing data was not imputed, nor outliers excluded. Descriptive statistics were calculated after patients were stratified by sex and compared using student’s t test, chi-squared test, Wilcoxon Rank Sum test, or a non-parametric equivalent as dictated by the data. Univariate difference between groups that had a p < 0.1 was explored using regression analysis, to identify covariate-adjusted independent associations of each variable with sex. We limited regression covariates to the a priori specified explanatory variables of sex, and sequentially included potential confounders including age, Charlson Comorbidity Index score and/or surgery type (six categories) when the outcome event rate was sufficient to support it (one independent variable per 10 patients who experienced an event of interest). Model fit was judged using the Hosmer–Lemeshow goodness-of-fit test and Area Under the Receiver Operating Characteristics curve for logistic regressions and R^2^ and residual vs. fitted plots for linear regressions. This post-hoc analysis had 80% power to detect a 6% difference in proportions and four unit difference in means with the available data. Analyses were performed using Stata software, version 15 (StataCorp Inc., College Station, TX).

## Results

### Included participants

There were 3506 patients screened for the EASE study between April 1, 2014 and July 31, 2015. In older patients, where data was available (*n* = 857), there were no statistically significant differences in EASE study exclusion rates between sex for transfers, nursing home residency, elective, trauma, palliative, and non-abdominal surgeries (p > 0.05). Older women were more likely than men to undergo conservative management as opposed to surgery (41% vs 34%, p = 0.03).

There were 322 elderly patients included in this post-hoc analysis from the EASE study. We were able to identify another 190 young patients from the EASE excluded cohort that met all inclusion criterion for the post-hoc analysis. Of the included 512 patients, there were 245 women (48%) and 267 men (52%), with a sex-ratio consistent with our overall excluded cohort. There were no differences in patient demographics or admission location type (p > 0.05). Women had a significantly more frequent diagnosis of cancer when compared to men (8% vs. 2%, p = 0.002; Table 1), which remained significant after adjusting for age (OR 4.12 [95% CI: 1.61–10.5], p = 0.003)

**Table 1:** Baseline characteristics.

	Females n = 245	Males n = 267	p-value
Age, years	62.4 (20.2)	64.7 (18.0)	0.2
Body Mass Index, kg/m2	26.8 (6.87)	27.4 (5.52)	0.3
Charlson Comorbidity Index†	0 (0–1)	0 (0–1)	0.4
ASA Physical Status Classification	2.49 (0.85)	2.49 (0.87)	0.9
Number of medications	3.9 (3.7)	3.9 (3.5)	0.8
Admission location *			0.7
Emergency room	213 (87)	234 (88)	0.8
Other Hospital transfer	26 (11)	30 (11)	0.8
Floor/Ward bed	6 (2)	2 (1)	0.1
Intensive care unit	0 (0)	1 (1)	0.3
Diagnosis*			0.2
Cholecystectomy/ Appendectomy	106 (43)	121 (45)	0.6
Intestinal obstruction	44 (18)	48 (18)	1
Hernia	31 (13)	48 (18)	0.1
Diverticulitis/Peritonitis/Ischemia	26 (10)	25 (9)	0.6
Cancer	20 (8)	6 (2)	0.002
Other surgery	18 (7)	19 (7)	0.9

Reported as mean (SD), unless indicated.

* n (%)

^†^ median (IQR)

### Preoperative and operative details

There were also no differences between sexes for perioperative outcomes, except that men were more likely than women to undergo hernia repair surgery (18% vs 9%, p = 0.001; Table 2), which remained significant after adjusted for age and Charlson Comorbidity Index (OR 2.33 [95% CI 1.35–4.02], p = 0.002). There were no differences in time to surgery or time spent in the operating room (p > 0.05).

**Table 2:** Sex stratified pre- and intra-operative outcomes.

	Females n = 245	Males n = 267	p-value
Time to surgical admission, hrs†	9.77 (6.28–14.4)	9.60 (5.55–13.5)	0.28
If Emergency room admit	10.3 (7.11–14.45)	10.08 (6.6–14.0)	0.44
If hospital transfer	4.51 (2.03–8.17)	3.07 (1.33–7.33)	0.65
If Floor/Ward bed	29.0 (7.01–74.5)	6.27 (4.65–7.89)	0.5
Time to surgery from index admission, hrs†	23.6 (12.6–50.0)	21.0 (11.2–50.0)	0.3
Time to surgery from surgery admission, hrs†	11.4 (2.23–41.1)	9.45(2.47–40.7)	0.9
Number of surgeries†	1 (1–1)	1 (1–1)	0.8
Number of surgeries*			0.9
1	229 (93)	251 (94)	0.8
2	13 (5)	11 (4)	0.5
3 or more	3 (1)	5 (2)	0.6
Surgery type*			0.02
Closed cholecystectomy/appendectomy	75 (30)	99 (37)	0.1
Open cholecystectomy/ appendectomy	30 (13)	25 (9)	0.2
Hernia repair	21 (9)	49 (18)	0.001
Small intestinal surgery	62 (25)	54 (20)	0.2
Colon surgery	35 (14)	27 (10)	0.1
Other surgery	21 (9)	13 (5)	0.09
Time in surgery, min†	101 (71–137)	104 (77–142)	0.3
Disposition after surgery*			0.4
Ward	214 (87)	242 (91)	0.2
Step down unit	5 (2)	1 (0)	0.08
Intensive care unit	26 (11)	24 (9)	0.5

Reported as mean (SD), unless indicated.

* n (%)

^†^ median (IQR)

### Post-operative differences between sexes

Women had higher rates of minor gastrointestinal complications when compared to men (55% vs 41%, p = 0.04; Table 3); this association was no longer significant after adjusting for age, Charlson Comorbidity Index and surgery type (OR 1.48 [95% CI: 0.91–2.39], p = 0.11). Men were more likely than women to experience major respiratory complications (9% vs. 3%, p = 0.02), which remained significant after adjusting for age (OR 2.72 [95% CI: 1.19–6.26], p = 0.02). There were no differences between sex with regards to overall mortality, hospital length of stay or discharge disposition (p > 0.05).

**Table 3:** Sex stratified post-operative outcomes.

	Females n = 245	Males n = 267	p-value
Patients who mobilized*	239 (97)	265 (99)	0.2
Time to mobilization, hours	35.0 (73.1)	33.1 (72.9)	0.7
Slow mobilizers (> 35 hours)*	60 (24)	52 (19)	0.1
Patients with foley catheter*	129 (53)	141 (52)	0.6
Days of foley catheter	6.26 (12.5)	5.55 (10.2)	0.6
Patient with total parenteral nutrition	43 (17)	42 (17)	0.6
Days of total parenteral nutrition	11. 7 (12.9)	9.38 (12.3)	0.4
Patient with any complication*	93 (38)	102 (38)	1
Major cardiovascular complication*	16 (7)	27 (10)	0.1
Major neurological complication*	35 (14)	43 (16)	0.6
Major gastrointestinal complication*	7 (3)	10 (4)	0.4
Minor gastrointestinal complication*	55 (22)	41 (15)	0.04
Major genitourinary complication*	3 (1)	6 (2)	0.5
Minor genitourinary complication*	17 (7)	19 (7)	0.9
Major intensive care complication*	2 (1)	6 (2)	0.3
Major infectious disease complication*	21 (9)	18 (7)	0.4
Minor infectious disease complication*	4 (2)	6 (2)	0.8
Major respiratory complication*	8 (3)	23 (9)	0.02
Major shock complication*	1 (0)	6 (2)	0.1
Major surgical complication*	7 (3)	12 (5)	0.3
Major unintentional injury complication*	11 (5)	10 (4)	0.6
Death	6 (2)	8 (3)	0.7
Number waiting to be discharged*	120 (49)	117 (44)	0.2
Time waiting for discharge, days†	1 (1–1)	1 (1–1)	1
Length of stay, days†	6 (4–12)	6 (3–10)	0.5
Discharge status*			0.6
Discharged home	183 (75)	204 (77)	0.7
Discharged with homecare	26 (11)	32 (12)	0.6
Discharged to subacute/rehab	21 (9)	16 (6)	0.3
Discharged to assisted living/long term care	8 (3)	6 (2)	0.5
Died in hospital	6 (2)	8 (3)	0.7

Reported as mean (SD), unless indicated.

* n (%)

^†^ median (IQR)

Major Complication = Clavian-Dindo Classification III or IV. Minor Complication = Clavian-Dindo Classification I or II. Waiting to be discharged = patient medically stable to be discharged from acute care but remains in-hospital due to lack of availability for community supportive care (example: subacute, rehabilitation or long term care)

## Discussion

Sex differences have been shown in many aspects of health care. Our study demonstrates that once surgical treatment was decided, there were little differences in perioperative and intra-operative care. Women had statistically greater cancer presentations whereas men had higher rates of hernia repairs. Similarly, there was no statistical difference in overall post-operative complications between men and women undergoing emergent surgical procedures, except for major respiratory complications, which were more likely to occur in men. Of note, older women appear to be conservative managed more often, compared to older men, when they present to an emergency surgery service. To our knowledge, no other studies have looked at sex differences exclusively in emergent general surgery.

Our findings are similar to a large Canadian observational study of almost 40,000 participants, where males had a significantly higher incidence of emergent surgery (32.5 vs 28.5%, p<0.001) [24]. Unlike our study, however, Grewal and colleagues [24] also showed a higher mortality rate in men postoperatively. The authors hypothesized that these sex differences may be due to higher preoperative risk factors secondary to a societal tendency for males to have fewer contacts with primary care, resulting in surgery at a later and more urgent stage of disease [24].

When gender bias was investigated at the primary care referral process, physicians themselves may be favoring the selection of men, as observed in elective knee and hip arthroplasties [25, 26], where men are 4 times more likely to be referred to surgery. Similar trends have been noted in cardiovascular surgery and critical care despite similar severity in illness [10]. These discrepancies warrant further exploration to determine if the sex of the most responsible physician or differing disease presentations of either sex plays a greater role when deciding upon surgical intervention.

Several social reasons have been identified as to why women are having less aggressive surgical intervention compared to men. Women themselves may have a decreased willingness to undergo surgery [25], due to the fear of post-operative risk and burden on family [27]. This may explain why women wait later for surgery and are then surgically found to have more advanced disease [28]. Our study noted that more women had gastrointestinal cancer requiring surgery compared to men. Similarly, Amri and colleagues [16] noted that despite a universal health care, women were still less often diagnosed through screening means and more often urgently, both which have been identified as risk factors for worse outcomes [29].

In non-emergent surgeries, a US nationwide retrospective data analysis in gastrointestinal surgery has shown that women tend to have lower mortality then men, and that this particularly pronounced in those 50 years or less [30]. Females also have lower mortality rates compared to men when trauma databanks have been analyzed [31, 32]. Physiologically it is hypothesized that higher levels of estrogen may lower inflammatory responses post trauma [33], which has also been hypothesized to explain why younger [1], but not older women [34, 35] have lower rates of mortality in ICU settings. Moreover, long-term (6–12 months) functional decline has been shown to be higher in females [36]. In addition, men had higher respiratory complications, including acute lung injury or respiratory failure, which is consistent with other studies [37]. However, even in surgical biomedical research, there is a clear disparity and sex bias that exists in which conclusions derived from studies based on predominately male subjects are overgeneralized [38]. In the past, women have been systematically left out of medical trials, impacting our lack of understanding about of female symptomatology, risk factors and pathology [14].

Limitations of our study include the over-representation of elderly patients given the nature of the EASE study, bias introduced by doing a post-hoc assessment, inability to adjust for confounding for the majority of our univariate comparisons because of low outcome event rates, as well relatively short follow-up time which was limited to hospital admission. As all non-operative patients were excluded from further data collection, we are unable to further explore important social selection biases in the selection of surgery patients. In the future, using larger administrative data bases would be ideal to further investigate these differences, although this may make specific outcomes unavailable. Our data may not be generalizable to other countries that do not have universal health care. However, even adjusting for insurance, age and diseases severity, US data from California suggests that women tend to receive less procedures [9], highlighting that, societal biases exist in addition to the physiological differences [13].

Strengths of our study include the relatively large sample size given the prospective study design, the wide age range including elderly, as well as the extensive data on comorbidities, intra and postoperative surgical details that was collected during study enrollment.

In summary, in this prospective study of EGS patients we found that women were more likely to be treated conservatively with non-operative approaches when presenting to an acute surgical service. However, once an operative approach was decided there were no sex differences with regards to perioperative care, overall mortality, hospital length of stay or discharge disposition. This study supports that the EGS model does not have sex biases once an operative approach is chosen. However, further work is necessary to explore potential sex biases in patients who are considered for operative verses non-operative management in EGS. These biases may disadvantage women in receiving inadequate care.
